# The Influence of Body Movements on Children’s Perception of Music with an Ambiguous Expressive Character

**DOI:** 10.1371/journal.pone.0054682

**Published:** 2013-01-24

**Authors:** Pieter-Jan Maes, Marc Leman

**Affiliations:** IPEM-Dept. of Musicology, Ghent University, Ghent, Belgium; UNLV, United States of America

## Abstract

The theory of embodied music cognition states that the perception and cognition of music is firmly, although not exclusively, linked to action patterns associated with that music. In this regard, the focus lies mostly on how music promotes certain action tendencies (i.e., dance, entrainment, etc.). Only recently, studies have started to devote attention to the reciprocal effects that people’s body movements may exert on how people perceive certain aspects of music and sound (e.g., pitch, meter, musical preference, etc.). The present study positions itself in this line of research. The central research question is whether expressive body movements, which are systematically paired with music, can modulate children’s perception of musical expressiveness. We present a behavioral experiment in which different groups of children (7–8 years, N = 46) either repetitively performed a happy or a sad choreography in response to expressively ambiguous music or merely listened to that music. The results of our study show indeed that children’s perception of musical expressiveness is modulated in accordance with the expressive character of the dance choreography performed to the music. This finding supports theories that claim a strong connection between action and perception, although further research is needed to uncover the details of this connection.

## Introduction

According to the theory of embodied cognition, human experiences are shaped by sensory-motor interactions with phenomena and objects in our environment [1], [2]. Music can be considered as an example of a complex and rich phenomenon that shapes experiences through sensory-motor interaction. Music is typically composed of sound traces that result from the manipulation of musical instruments and the sensory-motor interaction of people with these sound traces is considered as an important aspect of people’s engagement with music [3]–[5]. Sensory-motor interaction is not only important for the production of music, but also for the perception of music. It is commonly known that listening to music (i.e., perception) often induces spontaneous body movements (i.e., action). Studies have shown that these music-induced body movements generally reflect structural and expressive aspects of the music [6]–[9]. Only recently, there is growing evidence suggesting that the link between perception and action is bidirectional, in the sense that actions can modulate auditory perception as well. For example, studies have shown that the observation of a musician’s actions can influence people’s perception of pitch-related features [10], tone duration [11], and various structural parameters related to musical expression [12]. Moreover, studies have shown that movements which are performed concurrently with music or simple sound sequences can, at least momentarily, modulate people’s musical preferences [13] and their perception of musical meter [14], [15]. Also, Repp and Knoblich [16] have demonstrated that body movements (i.e., left/right movements) can similarly influence pitch perception (i.e., decreasing/increasing pitch) as a result of existing links between these motor and auditory representations acquired during extensive piano training.

In line with this last set of studies, we conducted a behavioral study to investigate whether expressive body movements can influence children’s perception of musical expressiveness. To this end, we applied a method that shows resemblances with the psychological principle of ‘evaluative conditioning’. Evaluative conditioning involves a process in which a person’s affective response or attitude (i.e., evaluation) towards a neutral stimulus (i.e., conditioned stimulus, CS) is changed due to a repeated pairing of that conditioned stimulus with another, affect-laden stimulus (i.e., unconditioned stimulus, US). Related to our study, the CS can be considered to correspond with the music, and the US to expressive body movements. We hypothesized that when children repeatedly perform expressive movements to music, their perception of the expressive character of that music will change in accordance with the expressive character of the body movements.

To test this hypothesis, we selected two musical excerpts with an ambiguous expressive character. The musical excerpts were ambiguous in the sense that the music can be perceived as either happy or sad due to a mix of musical cues that can be related to the expression of either happiness or sadness. Research has indicated that such music can elicit simultaneously mixed emotions of happiness and sadness in the listener [17], [18]. According to our hypothesis, the expressiveness of body movements, which children repeatedly perform while listening to such music, could later on “disambiguate” their perception of musical expressiveness towards one of both expressions contained in the music.

**Figure 1:**
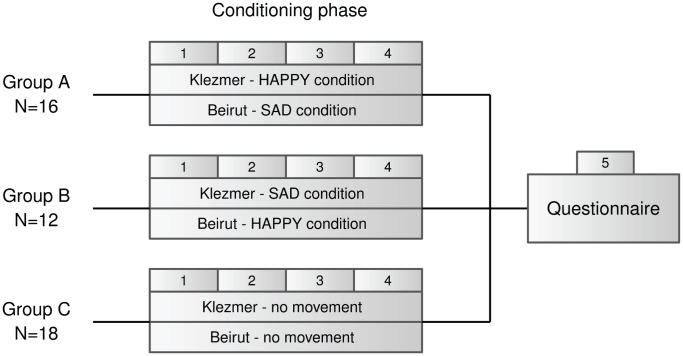
Schematic representation of the experimental design.

We developed two dance choreographies, explicitly distinct in expressive character (HAPPY choreography and SAD choreography) and adjusted to both musical excerpts used in the experiment. Two experimental groups (group A, 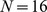; group B, 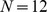) and one control group (group C, 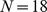) of 7–8 year old children participated in the experiment. By imitating a dance teacher, the experimental groups learned how to perform both dance choreographies. The learned dance choreographies were different between groups per musical excerpt, and counterbalanced per group between musical excerpts (see Fig. 1). During several sessions organized on four consecutive days, the choreographies were systematically repeated in order that a strong association would be developed in the children between the musical excerpts and the corresponding expressive dance choreographies. In what follows, the HAPPY condition relates to the condition during which a musical excerpt was associated with the HAPPY choreography, and vice versa, the SAD condition relates to the condition during which a musical excerpt was associated with the SAD choreography. The control group merely listened to both musical excerpts without performing or observing any body movement (i.e., CONTROL condition). Afterwards, the perception of musical expressiveness was assessed for all children by means of a questionnaire. In this study, we defined the “perception of musical expressiveness” in terms of how children would associate the music with concepts expressive of the categories *valence* and *arousal*. Valence describes the intrinsic attractiveness (positive valence) or aversiveness (negative valence) of the expression, while arousal describes the activation level of the expression (low versus high). The HAPPY choreography consisted of body movements that expressed a positive valence and a high arousal, while the SAD choreography expressed a negative valence and a low arousal. The questionnaire consisted of a list of bipolar adjective scales of which each scale consisted of one adjective that expressed a valence and/or arousal that matched the valence and/or arousal of the HAPPY choreography, and one that related to the SAD choreography. The hypothesis was then that children in the HAPPY condition would be more attracted to the adjectives with a positive valence and/or high arousal and vice versa.

## Methods

### Ethics Statement

Approval of an ethics committee is only obligatory according to the Belgian Law for experiments aiming towards research performed in view of the development of biological or medical knowledge (cf. ‘*7 May 2004 Law concerning experiments on the human person*’ (*Ch.II, Art.2, 11°*; *Ch.VIII, Art.11, 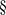
1*). Thus, no ethics approval was obligatory for the presented study. However, the ‘*12 February 2007 Advice no.40 related to the 7 May 2004 Law concerning experiments on the human person*’ advises that research should be meaningful and not harming the privacy of the participants, and that the participants provide informed consent for participation. Therefore, we obtained a written consent from the parents of the children participating in the experiment in which parents declared (1) that their children participated freely in the experiment, (2) that they were informed about the procedures and tasks inherent to the experiment, (3) that they got the chance to ask questions and that they received satisfying answers on these questions, and (4) that they agreed that video material was recorded for scientific and educational purposes only. Also, the data were processed and analyzed anonymously.

### Participants

The experiment was conducted with children of 7–8 years old (i.e., second year of primary school). Children of this age group have developed the appropriate cognitive-emotional basis for the tasks inherent to the present study. Studies have demonstrated that this age group is already able to identify basic musical emotions. The identification of the musical emotions happiness and sadness is most easy [19], and tempo and mode seem thereby important musical cues [20]–[22]. Next, research has shown that 7–8 year olds are able to synchronize movement with music, and with other actors [23], [24]. Also, research has demonstrated that they are able to decode and encode emotional meaning in expressive body movements [25], [26]. Moreover, in general, children of the second grade of primary school did not yet receive formal music education and they are less likely to have formed prior cognitive appraisals of expressiveness concerning the musical excerpts used in the experiment [27]. Finally, as 7–8 year olds are supposed to read properly, we could use a questionnaire based on bipolar adjective scales to rate the children’s perception of musical expressiveness.

We selected three separate classroom groups of children (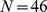) in two different schools. Both schools belonged to the same school system/network (in Dutch, ‘Vrije Basisschool’) and adhered to the same curriculum. Therefore, it was unlikely that the difference in schools imposed a considerable confound on the obtained results. Two classroom groups (of the same school) functioned as two separate experimental groups, the other (of another school) as control group. In the control group, no movement was conditioned in response to the music. The children only listened to the music. A first experimental group (i.e., *classroom group A*) consisted of 16 children (8 male, 8 female), a second experimental group (i.e., *classroom group B*) consisted of 12 children (7 male, 5 female), and the control group (i.e., *classroom group C*) consisted of 18 children (7 male, 11 female). We also tested the level of Extroversion of each child as we assumed it to be a factor that could have had an influence on the children’s ratings of the expressiveness they perceived in the music. Therefore, we used the Dutch translation of the Big Five Inventory test which is suited for children of the age group 7–8 years [28]. The test provided for the measure Extroversion a score from 1 to 5, with 1 as being ‘very introverted’ and 5 as being ‘very extroverted’. The questions were rated by the school teachers of the respective classroom groups. The validity of teachers’ ratings has been shown by Mervielde and colleagues [29]. For classroom group A, the mean was 3.70 (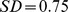), for classroom group B, the mean was 3.74 (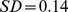), and for classroom group C, the mean was 3.35 (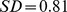). As such, we could say that all groups are relatively homogenous on the factor Extroversion.

### Materials

#### Musical excerpts

For the experiment, we selected two musical excerpts so that both experimental groups could be conditioned with both the HAPPY and SAD choreography (see Sect. [Dance choreographies]). In the selection of the musical excerpts, three main preconditions were taken into account. First, the musical excerpts had to be “real” music. Second, it was required that both the HAPPY and SAD choreography could be performed to both musical excerpts, and third, they had to be ambiguous in expressive character. Therefore, we searched for music with conicting happy and sad cues [17], [18]. As a first musical excerpt, we selected *Klezmer Csardas*, performed by *The Franz Liszt Chamber Orchestra* (Roby Lakatos) and recorded on the album *Klezmer Karma* (2006). In what follows, we will refer to this musical excerpt as the Klezmer excerpt. The second musical excerpt that was used was the song *Rhineland (Heartland)* from the album *Gulag Orkestar* (2005) of the band Beirut. Both musical excerpts had a duration of about 2 m 30 s. To pinpoint the expressive ambiguity of both musical excerpts, we analyzed music-technical properties in relation to the expressive character they had [17], [18]. For the Klezmer excerpt, we found that the aloof violin, the specific *Mi Sheberach* or altered Dorian mode (resembling the key in A minor), and several chromatic descending lines all contributed to a sad expressiveness. On the other hand, the Klezmer excerpt can also be interpreted as a joyful piece since percussion, syncopations, upbeats, and pizzicato by the cello created an instigating rhythm and dynamics. Moreover, Dmitri Shostakovich described Jewish folk music, to which the Klezmer genre belongs, in general as multifaceted as it can appear to be happy while it is tragic: “it’s almost always laughter through tears” [30]. In line with the Klezmer excerpt, the Beirut excerpt also begins in A minor. After the introduction, a modulation from A minor to G minor is noted. The minor key and slow tempo contributed to a feeling of sadness, whereas the instigating cadence extended by percussion might be responsible for a joyful feeling and may stimulate dancing.

It was investigated whether children of the control group rated the two musical excerpts differently. Therefore, for each scale, a statistical comparison was executed (i.e., Mann-Whitney U tests) between the children’s rating of the Klezmer excerpt and their rating of the Beirut excerpt. The results showed no significant differences (p>:05) between children’s ratings of both musical excerpts on any of the 14 bipolar adjective scales (see Sect. [Materials]).

#### Dance choreographies

We created two small dance choreographies that could be performed to both musical excerpts. Both choreographies had an opposite expression, namely *happiness/joy* (i.e., HAPPY) and *sadness/grief* (i.e., SAD). These expressions can be linked to the categories of valence and arousal. In the case of HAPPY and SAD, both categories are opposites; HAPPY refers to a positive valence and high arousal, while SAD reects a negative valence and low arousal. The general movement features characterizing the choreographies are derived from research pinpointing the expressive meaning of specific body movements [26], [31]–[34]. According to these studies, *happiness* or *joy* are portrayed by rotating movements around the vertical axis, upright dancing, fast pace, expansive movements, dynamic tension, high movement dynamics, jumping, etc. *Sadness* or *grief* are reected in low tension, slow pace, passivity, head down, contracted/collapsed upper body, etc. All these movement features were integrated into the dance choreographies. Also, there was a social aspect in the sense that in the HAPPY choreography, there were passages where children could move around each other freely using the complete dance (cf. social) space, whereas in the SAD choreography, children were more isolated from each other on specific spots in the dance space.

We wanted to provide quantifiable measures for the movement properties used in the choreographies which are indicated in previous research (see above) as corresponding to the expressions of happiness (i.e., positive valence and high arousal) and sadness (i.e., negative valence and low arousal). Therefore, we recorded a performance of the two choreographies executed by the dance teacher (see Sect. [Procedure]). Movements of the chest and the dominant hand were considered as being most informative in conveying the overall expressiveness of the dance choreographies. Three-dimensional positions of these body parts were recorded at a sample rate of 100 Hz with an OPTITRACK infrared optical system consisting of 12 synchronized cameras with related ARENA motion capture software (http://www.naturalpoint.com). Based on this data, we calculated various movement features which are summarized in Table 1. The results indicate that, in the HAPPY choreography, movements were faster, more accelerated, more impulsive, and higher, the upper limbs were more expanded, there was more rotation of the body around the vertical axis, and more distance was covered in the horizontal direction as well as in the vertical direction compared to the SAD choreography. These results confirm, in a quantitative manner, that the HAPPY as well as the SAD choreography are characterized by an expressiveness which is shown to be related with happiness (i.e., positive valence and high arousal) and sadness (i.e., negative valence and low arousal).

**Table 1:** Summary of the movement features extracted from the SAD and HAPPY choreography with the statistics of the differences between both (where possible).

	*U*	*z*	*p*	*r*	*Mdn*	*Mdn*
					SAD	HAPPY
Velocity-chest (m/s)	45×10^6^	−51.16	<.01	.33	0.33	0.53
Velocity-hand (m/s)	20×10^6^	−96.77	<.01	.62	0.51	1.80
Acceleration-chest (m/s^2^)	53×10^6^	−34.54	<.01	.22	0.55×10^−2^	0.89×10^−2^
Acceleration-hand (m/s^2^)	35×10^6^	−67.24	<.01	.43	0.94×10^−2^	0.03
Height-chest (m)	32×10^6^	−74.41	<.01	.48	1.21	1.24
Height-hand (m)	39×10^6^	−59.76	<.01	.39	0.81	0.98
Rotational speed (deg/sec)	30×10^7^	−66.10	<.01	.27	14	44
Impulsiveness-hand	116	−6.58	<.01	.74	0.01	0.05
Distance between hands (m)	44×10^6^	−50.81	<.01	.32	0.70	0.78
Covered horizontal distance-chest (m)	21.01 (SAD)			26.58 (HAPPY)		
Covered horizontal distance-hand (m)	34.59 (SAD)			95.52 (HAPPY)		
Covered vertical distance-chest (m)	4.77 (SAD)			18.95 (HAPPY)		
Covered vertical distance-hand (m)	9.85 (SAD)			54.6 (HAPPY)		

#### Questionnaire

The questionnaire was used to assess the childrens’ perception of musical expressiveness. *“The perception of musical expressiveness”* relates, in this study, to how children perceive music as being expressive of the qualities valence and arousal. In other words, it relates to how children attribute positive/negative valence and/or high/low arousal to a musical excerpt. In our study, we hypothesized that this perception is a function of the expressiveness of the body movements that children associated with the music. Therefore, we expected that children in the HAPPY condition would be more attracted towards a perception of the music as having positive valence and/or high arousal and, in contrast, that children in the SAD condition would be more attracted towards a perception of the same music as having negative valence and/or low arousal.

In the questionnaire, we used a method based on semantic differential scales. The semantic differential method was introduced by Osgood and colleagues [35] as a technique for measuring the various dimensions of a person’s meaning attributed to an object or concept. According to the procedures of this method, individuals are presented with pairs of bipolar (opposite) adjectives placed each at one end of a continuous rating scale (mostly a seven-point rating scale, although five-point scales are also used). These rating scales are considered to yield interval data allowing parametric analysis methods [36], [37]. Confronted with these scales, individuals are asked to rate the association of a particular concept or object in relation to the specific bipolar adjective pairs. Osgood and colleagues were interested in identifying some basic dimensions of connotative meaning which they found to be *evaluation* (i.e., positive-negative continuum), *potency* (i.e., strong-weak continuum), and *activity* (i.e., active-passive continuum). The first two dimensions resemble respectively the categories valence and arousal that were used in this study to define (1) the expressive movement choreographies and (2) the perception of musical expressiveness.

The technique has been used in a large body of studies to rate people’s perception of expressiveness in music [38]–[44]. In our study, we created a 14-item, five-point, semantic differential list to let children rate both musical excerpts (see Table 2). We opted for a five-point scale as a pilot study had indicated that children of age 7–8 years experienced difficulties using a seven-point scale. The adjectives we used express either positive valence (i.e., evaluation) and/or high arousal (i.e., potency), or negative valence and/or low arousal. As such, the expressive character of each adjective matched the expressive character of one of the choreographies (HAPPY or SAD). The validation of the adjectives was done in a pilot study in consultation with children of the same age as the ones participating in the experiment to ensure that the “vocabulary” would be understood by the children. As will be explained in more detail in the Results section, during the analysis of the semantic differential scales, the bipolar adjectives were structured as such that the ones expressing positive valence and/or high arousal are positioned to the right-hand side of the scale, and the ones expressing negative valence and/or low arousal are positioned to the left-hand side of the scale. Accordingly, the higher the score, the more the music was associated with adjectives with positive valence and/or high arousal, and vice versa. Of course, during the experiment, these positions were randomized to avoid biasing the responses as much as possible.

**Table 2:** Overview of the bipolar adjectives (translated from Dutch to English) used in the semantic differential scales.

	Bipolar adjectives	
1	miserable	gay
2	calm	busy
3	stagnated	motile
4	unsafe	safe
5	slow	fast
6	sad	happy
7	brute	kind
8	bleak	snug
9	grey	abloom
10	lonely	cosy
11	touching	cheerful
12	discontent	content
13	lazy	energetic
14	gloomy	merry

Additional to the semantic differential scales, we used the *Wong-Baker* FACES rating scale to assess in a visual way, the children’s perception of musical expressiveness [45]. At one end of that scale, a very happy emoticon face was represented, at the other end, a very sad emoticon face was represented, while in between, four faces evolved subtly from very happy to very sad. Originally, this rating scale was used to rate the perception of pain. However, the rating scale is suited to measure the perception of musical expressiveness of children in terms of HAPPY and SAD. Similar to the rating method of the semantic differential scales, the children where asked to indicate the face that according to them, could be associated best with the music. Also, while analyzing the results, the very happy face was given the highest score (i.e., 5) while the very sad face the lowest (i.e., 0). As such, we hypothesized that the children in the HAPPY condition, would have a significantly higher rating than the ones in the SAD condition.

### Procedure

The experimental procedure consisted of two separate parts (see Fig. 1). A first part comprised the conditioning phase, while during the second part, children had to rate the different musical excerpts by means of the questionnaire. The conditioning phase was subdivided into four sessions spread over four successive days (i.e., Monday to Thursday). On the first day, a female dance teacher (16 years of classical ballet training) instructed both dance choreographies to both experimental groups (i.e., classroom group A and B). Children were simply instructed to imitate the movements of the dance teacher in correspondence with the music. Thereby, she made sure not to use any verbal descriptions in terms of valence and arousal. The duration of this first session was about one hour. On day 2, 3 and 4, the experimental groups were two times presented with both musical excerpts. Supported by the dance teacher, who danced along, children had to perform the dance choreographies in correspondence with the musical excerpts. The order in which the musical excerpts were presented to the children was changed every next day. Each session had a duration of about 15 minutes (small breaks in between the dancing parts included) and were held in a large room equipped with a decent speaker system. For the control group (i.e., classroom group C), the same procedure was applied but instead of moving to the music, the children just listened to the music. That is to say, children got the instruction to listen to the music while sitting at a desk. This created a passive listening situation because it prevents children from having extensive movement responses to the music. These listening sessions were organized in the classroom where classes normally were given.

The second part of the procedure, during which children had to rate the musical excerpts by means of the questionnaire, was conducted on day 5 (i.e., Friday). This time slot of one day between the last conditioning session and the actual rating of the musical excerpts was implemented on purpose, to avoid that the movement performance itself biased certain responses to the semantic differential scales. The children filled in the questionnaire in the classroom where classes were normally given. To start off, the children were introduced, in group, with the semantic differential scale procedures. Therefore, we used images of human figures with obvious opposite physical features (e.g., slim-plump, old-young, etc.). Afterwards, they could ask questions if necessary. Finally, they had to listen to a musical excerpt, played in a loop, while filling in the questionnaire at their own tempo. If all children were ready, the same was done for the other musical excerpt. The order in which the musical excerpts were presented was changed for both experimental groups.

## Results

In this section, we provide the results related to our main research question, namely whether there is a statistical difference in children’s perception of the expressiveness of a musical excerpt in terms of valence and arousal as an effect of the expressiveness of the movements (i.e., SAD or HAPPY) that were conditioned in association with that musical excerpt.

**Figure 2:**
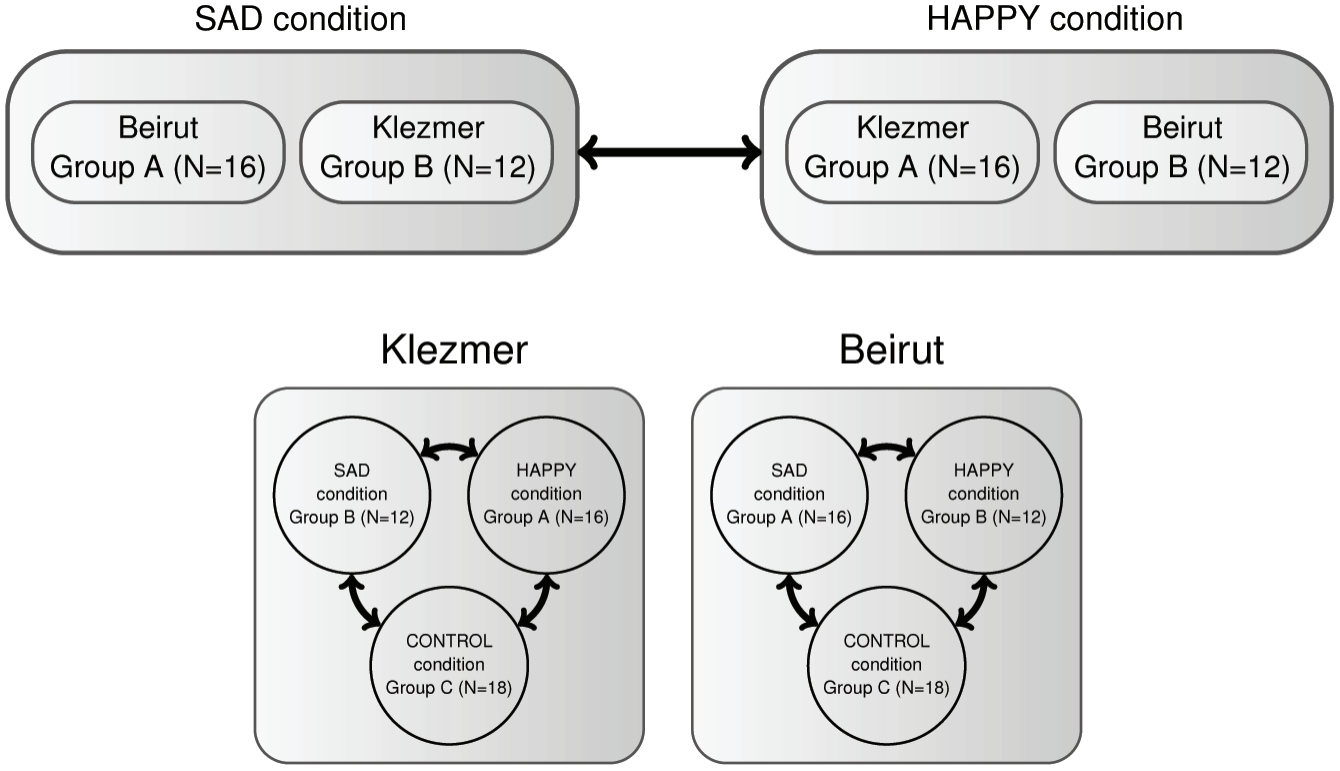
Schematic representation of the data structures used in the analysis.

In general, the method used to analyze the children’s ratings on the semantic differential scales consisted of three main parts. In the first part, as can be seen in Fig. 2 (top), we focused on the general differences between the HAPPY and SAD condition. Thereby, no differentiation was made between both musical excerpts. This was appropriate as the analysis of the data of the CONTROL condition showed no significant differences between the ratings related to both musical excerpts (see Sect. [Materials]). Based on this data structure, univariate differences (i.e., individual scales) between the conditions were tested statistically. In the second part, we applied - again departing from the same data structure - a multivariate principal component analysis (PCA) in order to reduce the multidimensional data set (i.e., 14 semantic differential scales) into a smaller number of informative principal components (PCs). In the third part, we statistically tested the differences and relationships between the different conditions (i.e., HAPPY, SAD, CONTROL) per individual musical excerpt (see Fig. 2, bottom).

As already mentioned in the Materials section, the bipolar adjectives of each scale were ordered as such that the ones expressing positive valence and/or high arousal were positioned to the right-hand side of the scale, and the ones expressing negative valence and/or low arousal were positioned to the left-hand side of the scale. As such, the higher the score, the more the music was associated with adjectives representing positive valence and/or high arousal, and vice versa.

Also, in advance of the actual analysis, we calculated Cronbach’s alpha to measure the internal consistency of the responses on the different scales. A Cronbach’s alpha of.87 indicated that the questionnaire had a good internal consistency (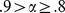) and was as such a reliable tool.

### Part 1: Univariate Analysis

**Figure 3:**
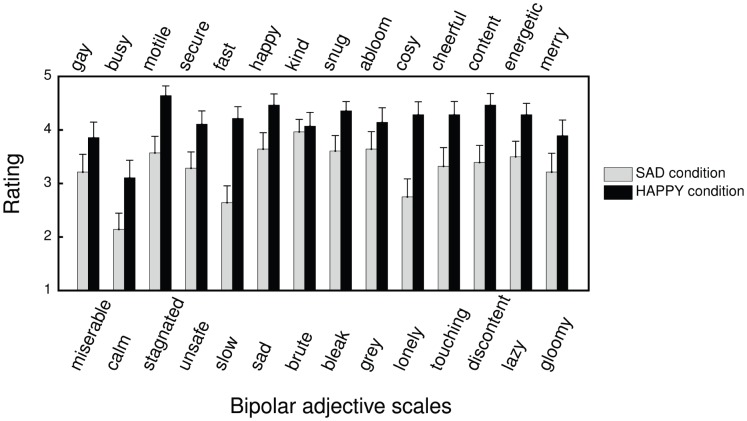
Representation of the mean (with standard error bars) of the ratings related to each semantic differential scale for the HAPPY condition (N = 28) and SAD condition (N = 28).

In Fig. 3, a descriptive overview is given of the average rating (and standard error) per scale, per condition. In the first part of the analysis, we performed a univariate statistical analysis on these 14 different bipolar adjective scales (see Table 2). Nonparametric Mann-Whitney U tests were applied (as normality of the corresponding distributions was systematically violated) in order to investigate, for each scale, whether there was a significant difference in rating between the SAD condition and HAPPY condition. Given the fact that we consider specific scales, rather than the set of scales as a whole, no correction for multiple comparisons (e.g., Bonferroni) was necessary [46].

As can be seen in Table 3, a significant difference between the conditions was found (with 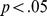, at least) for 8 of the 14 scales (i.e., *calm-busy*, *stagnated-motile*, *unsafe-safe*, *slow-fast*, *sad-happy*, *lonely-cosy*, *discontent-content*, *lazy-energetic*).

**Table 3:** Results of the statistical Mann-Whitney U tests indicating the differences in rating on each semantic differential scale (see Fig. 2) between the SAD condition (N = 28) and the HAPPY condition (N = 28) with scale 1 = miserable-gay, 2 = calm-busy, 3 = stagnated-motile, 4 = unsafe-safe, 5 = slow-fast, 6 = sad-happy, 7 = brute-kind, 8 = bleak-snug, 9 = grey-abloom, 10 = lonely-cosy, 11 = touching-cheerful, 12 = discontent-content, 13 = lazy energetic, and 14 = gloomy-merry.

Scale	1	2	3	4	5	6	7	8	9	10	11	12	13	14
*Mdn*-SAD	3.5	1	4	3.5	2	4	4	4	5	2	3.5	4	4	4
*Mdn*-HAPPY	5	4	5	5	5	5	5	5	5	5	5	5	5	5
*U*	322	276	241	279	193	271	362	300	331	202	288	242	279	313
*z*	−1.24	−2.01	−2.91	−1.96	−3.41	−2.27	−.53	−1.63	−1.12	−3.35	−1.85	−2.75	−1.98	−1.43
*p*	.225	.046	.003	.049	.001	.023	.611	.104	.264	.001	.065	.006	.047	.155
*r*	.17	.27	.39	.26	.46	.30	.07	.22	.15	.45	.25	.37	.26	.19

Supplementary to the analysis of the semantic differential scales, we performed a Mann-Whitney U test to check whether there was a statistical difference between the conditions related to the ratings of the Wong-Baker rating scale. The results indicated that children in the HAPPY condition (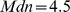) rated significantly higher (i.e., associate the music with more happy faces) than in the SAD condition (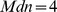), 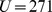, 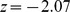, 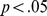 (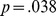), 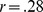.

### Part 2: Multivariate Analysis (PCA)

The individual univariate analyses showed that significant differences existed on 8 of the 14 bipolar adjective scales and that a same tendency could be observed for the other scales. With a PCA, we wanted to investigate to what extent these between-group differences on all bipolar adjective scales (i.e., 14-dimensional semantic space) could be reduced to between-group differences on a lower number of underlying, interpretative dimensions. As the data set did not meet the assumption of multivariate normality due to the bimodal nature of the data (i.e., each group will be attracted to a different end of a bipolar adjective scale), no inferential statistics could be invoked regarding the underlying population [47]. However, a PCA can provide valuable descriptive information for our data set.

The results of the PCA (with additional clockwise factor rotation of 30°) reveal that the first two principal components (PCs) explain respectively 39.19% and 13.36% of the total variance. The factor loadings, representing the magnitude and sign of each scale’s contribution to the first two PCs, are given in Table 4. Inspecting these factor loadings, one observes that the factors (i.e., scales) contributing most to PC1 are explicitly valence related, while the scales related to arousal (indicated in grey) have only a low contribution. The positive (right) direction of the axis is then positive valence, the negative (left) direction negative valence. Vice versa, scales related to arousal contribute explicitly to PC2, while valence related scales have only a low contribution. The positive (vertical upward) direction of the axis is then high arousal, the negative (vertical downward) direction low arousal. As such, we can interpret the most important components underlying the 14-dimensional semantic space as representing the categories of valence and arousal.

**Table 4:** Loadings related to the first two PCs obtained from the PCA of the 14 semantic differential scales.

Loading PC1	Adjective	Loading PC2	Adjective
.4412	abloom	.4985	busy
.3693	cheerful	.4708	fast
.3478	cosy	.3726	energetic
.3320	content	.3156	motile
.3295	secure	.2818	merry
.2963	snug	.2509	gay
.2726	kind	.2001	grey
.2589	gay	.1864	brute
.2151	happy	.1795	happy
.2125	merry	.1251	touching
.0784	energetic	.1013	content
.0331	slow	.0820	anxious
.0252	motile	.0680	bleak
.0184	calm	.0663	cosy
−.0184	busy	−.0663	lonely
−.0252	stagnated	−.0680	snug
−.0331	fast	−.0820	secure
−.0784	lazy	−.1013	discontent
−.2125	gloomy	−.1251	cheerful
−.2151	sad	−.1795	sad
−.2589	miserable	−0.1864	kind
−.2726	brute	−.2001	abloom
−.2963	bleak	−.2509	miserable
−.3295	anxious	−.2818	gloomy
−.3320	discontent	−.3156	stagnated
−.3478	lonely	−.3726	lazy
−.3693	touching	−.4708	slow
−.4412	grey	−.4985	calm

**Figure 4:**
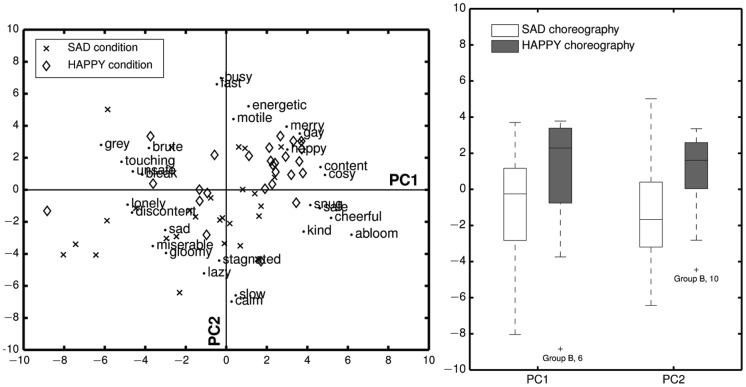
Left: Representation of the component scores (diamonds and crosses) and (scaled) loadings obtained from the PCA. Right: boxplots indicating the distributions of the component scores on the first two PCs.

In a next step, we divided the component scores, representing the scores of the children on the first two PCs, in two groups (see Fig. 4). One group consisted of the scores corresponding to the HAPPY condition (Fig. 4, diamonds) and one group consisted of the scores corresponding to the SAD condition (Fig. 4, crosses). As explained, we could not invoke inferential hypothesis testing to investigate the between-group differences. However, the descriptive statistics for the sample used in the experiment indicated that the mean (1.25, SD = 2.97) and median (2.28, IQR = 4.10) valence are higher (i.e., more positive) for the HAPPY condition than the mean (−1.25, SD = 3.22) and median (−0.25, IQR = 4.00) valence of the SAD condition. Similarly, the mean (1.10, SD = 1.91) and median (1.61, IQR = 2.55) arousal are higher for the HAPPY condition than the mean (−1.10, SD = 2.72) and median (−1.66, IQR = 3.59) arousal of the SAD condition.

### Part 3: Individual Musical Excerpts

**Figure 5:**
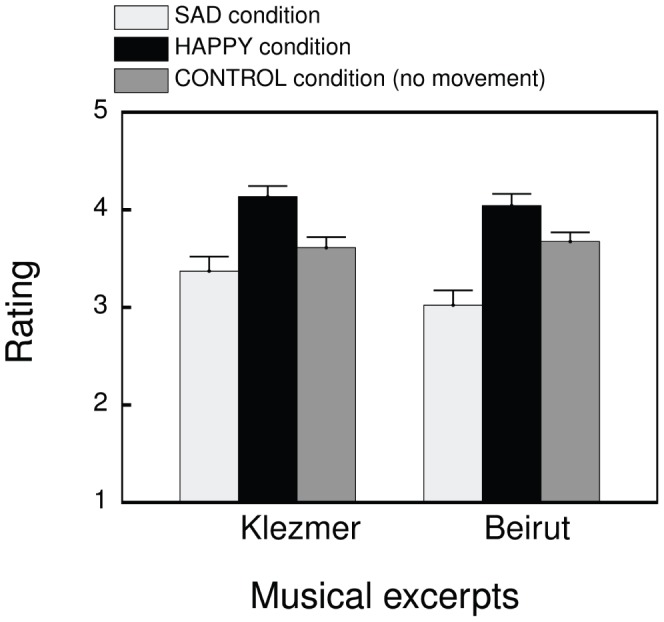
Representation of the mean (with standard error bars) of the ratings related to all 14 semantic differential scales per condition, and per musical excerpt.

In the third part of the analysis, we investigated statistical differences and relationships between the HAPPY, SAD and CONTROL condition for each individual musical excerpt. The children’s responses to the 14 scales were averaged as to obtain one value per respondent [36], [37]. A descriptive overview of the resulting data set can be addressed in Fig. 5. Because the assumption of normality was violated, we applied nonparametric Kruskal-Wallis tests in order to evaluate whether there is a statistically significant difference between the HAPPY, SAD and CONTROL condition. A Kruskal-Wallis test is the nonparametric analogue of a one-way ANOVA to test whether there are significant differences between the mean ranks of three or more independent samples. Moreover, we applied a follow-up test, called the Jonckheere test, to investigate whether the mean ranks significantly increased from the SAD condition to the CONTROL condition to the HAPPY condition. In other words, a Jonckheere test enabled us to investigate whether a trend existed across conditions. We expected an ascending trend.

For the Klezmer excerpt (see Fig. 5, left), the Kruskal-Wallis test indicated that the rating of perceived musical expressiveness was significantly affected by the conditioned movement response (i.e., the mean ranks of the different conditions are significantly different), 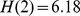, 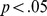 (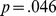). Moreover, a Jonckheere test revealed a significant trend in the data: from SAD condition to CONTROL condition to HAPPY condition, the mean ranks increased, 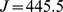, 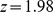, 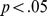 (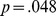), 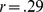.

Also, for the Beirut excerpt (see Fig. 5, right), a Kruskal-Wallis test indicated that the rating of perceived musical expressiveness was significantly affected by the conditioned movement response, 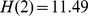, 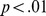 (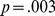). Again, a Jonckheere test revealed a significant trend in the data: from SAD condition to CONTROL condition to HAPPY condition, the mean ranks increased, 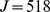, 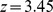, 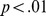 (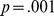), 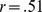.

## Discussion

The main research question of this study was whether a conditioned association between an expressively ambiguous musical excerpt and expressive body movements (defined in terms of valence and arousal) has an effect on children’s perception of musical expressiveness (in terms of valence and arousal). Children’s perception of musical expressiveness has been assessed by means of the semantic differential method [35]. Univariate analyses of the individual scales revealed significant differences on 8 of the 14 scales. More in particular, in the HAPPY condition, children perceived the music as being significantly more *busy*, *motile*, *safe*, *fast*, *happy*, *cosy*, *content* and *energetic* compared to the SAD condition. In general, a same tendency was observed for the adjectives *gay*, *brute*, *snug*, *abloom*, *cheerful* and *merry* although the difference were not statistically significant. Next to this, the results of the children’s rating on the *Wong-Baker FACES* rating scale show that they associated the musical excerpts with happier faces in the HAPPY condition than in the SAD condition. These results indicated that the adjectives as well as the emoticons that children associated with music matched the expressiveness of the movements that were conditioned. The fact that the same tendency is observed for all variables provided strong support for our original claim that conditioned body movements in response to music can have an effect on how musical expressiveness is perceived.

Next, the descriptive findings that resulted from a multivariate PCA suggested that the between-group differences found in the univariate analyses, could be reduced to between-group differences on the dimensions valence and arousal. The children in the HAPPY condition perceived the music as being more positive and higher in arousal, matching exactly the expressive qualities of the corresponding conditioned body movements.

Finally, the results related to the ratings in the HAPPY, SAD and CONTROL condition in response to the individual musical excerpts indicated, for both musical excerpts, a significant effect of the conditioned movement response (i.e., there were significant differences between the conditions). Moreover, a significant trend was found in the data, signaling an increase in rating value from the SAD condition to the CONTROL condition to the HAPPY condition. Again, as higher ratings corresponded with the expressive qualities of the HAPPY choreography and vice versa, we could observe a match between the expressiveness of the conditioned movements and the perception of musical expressiveness.

In conclusion, an in-depth analysis of the questionnaire data revealed, in general, a significant effect of a conditioned association between music and expressive body movements on the perception of musical expressiveness. However, considering the results of the ratings of the HAPPY, SAD, and CONTROL condition in response to both musical excerpts, we could observe that more clear differences between the conditions occurred with the Beirut excerpt compared to the Klezmer excerpt. A plausible explanation for this observation could be that it was more difficult to manipulate the perception of the Klezmer excerpt towards more negative and lower arousal qualities than it was the case for the Beirut excerpt (see Fig. 5). Research has shown that 6–8 year olds already associate fast tempi with happiness and slow tempi with sadness [22]. As the Klezmer excerpt had a relatively faster tempo than the Beirut excerpt, this could have been a reason why it was more difficult to condition the Klezmer excerpt towards a low valence rating, than the slower Beirut excerpt towards a high valence rating. Next to this we observed that it was more difficult in general to direct the perception towards negative valence and weak arousal (Fig. 4 (right) shows more spread in the data of the SAD condition compared to the data of the HAPPY condition). It has been suggested in previous research that rhythmic engagement and coordination with music in a social context may be a source of positive affect [48], [49]. It is therefore not unlikely that this effect interfered, at least partly, with the effect imposed by the conditioning procedure. As a result, the conditioning of body movements expressive of sadness in relation to the music would be expected to be more difficult, while the conditioning of body movements expressive of happiness would benefit from this aspect of social interaction.

Previous studies have suggested that body movements performed to music or simple sound sequences can have an influence on the perception of structural features, such as rhythm and pitch [14]–[16]. Also, it has been shown that body movements can influence people’s musical preferences [13]. The results of our study complement these findings by showing that, when an expressive dance is systematically paired with music with an ambiguous expressivity, the music can acquire the emotional meaning of the dance (at least for a few days). Given the fact that an experimental manipulation of music-movement associations resulted in corresponding effects on the perception of musical expressiveness, it is suggested that embodiment can influence children’s perception and interpretation of musical expressiveness. These results seem to support the embodied cognition theory [3], [4], which states that movements made in response to music can invoke an action semantics and that listening to music appeals to this semantics as a guide for meaning formation and the perception of musical expressiveness.

Several studies have pointed out that music can evoke specific expressive movements in a rather straightforward and unambiguous manner [6]–[9], suggesting that the link between movement and sound can be stable and conditioned by strong social/cultural and natural native forces. However, much of the world’s music evokes a less straightforward and a more ambiguous expressiveness, which leaves room for variation in the coupling of music to body movements. The question addressed in the present paper is whether some associations between music and expressiveness in actions can be conditioned at short term, so that underlying mechanisms of embodiment can be revealed. To cope with this, we used non-familiar musical excerpts with an ambiguous expressive character and we worked with children, as it is less likely that they have formed prior cognitive appraisals of expressiveness related to the musical excerpts [27]. However, the question remains whether our findings can be generalized to adults and music that evokes body movements in a straightforward, unambiguous manner. We argue, based on existing literature, that there is good reason to assume that conditioning processes are more generally involved in people’s engagement with music. Several theoretical accounts have advocated that listeners’ engagement with music relies, although not exclusively, on people’s ability to identify and simulate the effort, dexterity, touch, etc. of body movements from which the music originated [5], [8], [50], [51]. Importantly, it is suggested that this ability is developed, at least partly, through associative learning processes. Repeated experiences of body movements together with their auditory consequences create, after some time, a conditioned relationship between auditory and motor representations. As a consequence, when sounds are heard, associated motor representations are automatically and concurrently activated. This process is not limited to musical sounds and gestures [52]–[54] but is constantly on-going in our daily interactions with the world (e.g., walking, slamming a door, etc.). Moreover, as Godøy [8] mentions, “several instances of similar types of gestures with similar sensation of effort may be generalized into more generic types, or schemata” (or, what Cox [5] calls, ‘exertion schema’). In summary, if one assumes that motor simulation is a general aspect of listener’s engagement with music, conditioned relationships between sounds and body movements may be considered as an important, underlying aspect therein.

In the experiment, children learned the specific dance choreographies by imitating the movements of a dance teacher. Also, the children learned and performed the choreographies together, per group. This entails that other factors than the actual execution of body movements could have had an influence on the observed effect. First, there is the factor of mere visual observation of other’s body movements. Based on theories claiming that execution and observation of actions are represented in a similar manner [55]–[57], it could be argued that mere observation of body movements would result in similar results. Empirical support for this standpoint is provided by Thompson and colleagues [10] showing that merely watching a musician’s performance reliably influences perceptions of musical structure and affective interpretations of music. However, in contrast to these findings, studies of Phillips-Silver and colleagues [14], [15], investigating the effect of body movements on the perception of musical meter in ambiguous rhythmical patterns, showed that the actual performance of body movements without visual observation influenced the interpretation of musical meter while mere observation of the movements did not. However, it would be interesting to investigate into more detail the individual influences of performing and observing body movements on the perception of musical and affective properties of music. Second, on the basis of the presented design, it could not be distinguished whether it were aspects inherent to the performed body movements itself, aspects related to moving together with others in a social context, or the combination of both that established feelings of happiness and sadness. As Kirschner and Tomasello [48] have indicated, joint interaction of children in a social context (like dancing to music) can effectively create a positive collective experience, thereby “satisfying the intrinsic human desire to share emotions, experiences and activities.” Accordingly, it is likely that the effects resulting from children’s joint interaction may interfere, at least partly, with the effects promoted by the conditioning process. Thereby, it must be taken into account that these interference effects are possibly different according to the expressive nature (i.e., happy/sad) of the conditioned body movements. Therefore, it is necessary to further investigate the relationship between (1) the actual performance of expressive body movements (without the presence of others), (2) the mere visual observation of expressive body movements, and (3) the condition of moving together with others. In future research, the relationship performance/observation could be investigated by adding a condition in which a group of children merely observe expressive movements. The reverse (i.e., performance without visual observation) is more problematic as it is difficult to instruct a new dance choreography to children without visual observation (e.g., imitating a teacher). Verbal descriptions of expressive body movements could be an alternative for visual observation. The relationship individual performance/social performance could be investigated by adding an experimental group in which children perform the dance choreographies individually, without the presence of others.

Next to the factors ‘visual observation’ and ‘moving in a social context’, it cannot be excluded that the observed effects of the presented study are the result of other processes (e.g., biological, neurological, etc.) that accompany or underlay movement, instead of mere movement itself. This remark is similar to a remark Saint-Germier [58] made in reaction to some studies of Phillips-Silver and Trainor [14], [15]. In these studies, it was shown that body movements influenced the perception of metrical structures and rhythm. Saint-Germier agreed that body movements play a cognitive role, “in a way if you don’t take the body into account, then you miss a crucial part of the determinants of the cognitive processing”. However, based on findings of another study of Phillips-Silver and Trainor [59], he claimed that the influential role of body movements does not imply that body movements are *constitutive* for the observed effects (as the results of Trainor’s study showed that the stimulation of the vestibular system produces the same effects). The same remark can be applied to our study. However, although body movements might not be constitutive for the observed effect, they anyhow play a considerable role in the process in which music is connected to expressive concepts.
